# The Fallacy of Person‐Centred Care: Deconstructing the Discourse to Reimagine Practice

**DOI:** 10.1111/nup.70043

**Published:** 2025-10-07

**Authors:** Asam Latif, Nargis Gulzar

**Affiliations:** ^1^ Faculty of Medicine and Health Sciences University of Nottingham Nottingham England; ^2^ Midlands Pharmacy Team (Workforce, Training & Education) NHS England Leicester England

**Keywords:** deconstruction, healthcare professional, Nietzsche's philosophy, ‘person‐centred care’, resistance, Über‐professional, ‘will to power’

## Abstract

Person‐centred care dominates today's sociopolitical landscape, influencing the approach and conduct of healthcare institutions, organisations and practices. It seeks to elevate and transcend former biomedical models by centralising a person's needs, preferences and values in the care process. Positioned as the ‘gold standard’ approach, person‐centred care has become a central attribute in shaping professional identities and public discourse, influencing the ethos, attitudes and behaviours of healthcare professionals. Despite its dogmatic prominence in policy and professional discourse, there are entrenched bureaucratic structures, organisational barriers and conflicting agendas that impede professional efforts to uphold patient agency and autonomy; this has resulted in inconsistencies in its understanding and implementation. Furthermore, the framework itself fails to empower healthcare professionals to challenge practice when it is felt that person‐centred principles are compromised, rendering it little more than a rhetorical device used to promote self‐interest, enhance professional status and power. Given this fallacy, this critique contends that person‐centred care is effectively ‘dead’; its demise regrettably orchestrated at the hands of those entrusted to deliver it. In line with a Derridean deconstructive approach, we also provocatively question whether the very concept was ever ‘alive’ to begin with and whose interest it ultimately served. While its demise may signal time for a paradigmatic shift, it also presents an opportunity to reimagine healthcare practice in a manner that aligns with a more authentic approach. Inspired by Nietzsche's concept of the Übermensch (‘Over‐man’), we propose a vision of the ‘Über‐professional’, whose *‘Will to Power’* transcends institutional constraints and conventional practices. By embracing authenticity, the Über‐professional model offers both opportunity and ‘permission’ for adoptees to recognise and resist practices when these conflict with the provision of care. It therefore empowers them to ensure that all voices are heard, preferences are respected and the interests of patients are fully represented in all care decisions.

## 1 Introduction

In many healthcare settings, discourses on person‐centred care serve as the dominant sociopolitical narrative, shaping the fundamental values that guide nurses, doctors, pharmacists and other healthcare professionals in their approach and delivery to care (Nolte et al. 2020; Öhlén and Friberg 2023). To its proponents, person‐cantered care offers a holistic approach that emphasises respect, empathy and understanding to tailor care according to people's beliefs, values, unique needs and treatment preferences (McCance et al. 2011). Prevailing biomedical models are largely focused on diagnosing and treating disease based on biological factors (Zhao et al. 2016). In contrast, person‐centred care seeks to offer a holistic approach that encompasses agency, a multidimensional appreciation of the person's life including consideration of sociocultural contexts. In doing so, person‐centred care aspires to strengthen recipient participation through collaborative decision‐making in a bid to promote patient autonomy and empowerment. From this approach, the promise of a more balanced person‐professional relationship is formed, transforming people from being mere passive recipients to active partners in their care (Phelan et al. 2020). To improve organisational performance, person‐centred care principles are being adopted across healthcare systems, extending their influence on care culture as well as broader codesign and coproduction service‐development initiatives (Santana et al. 2018).

While the concept of person‐centred care may seem instinctively and morally commendable, its interpretation and application in practice has been persistently inconsistent (Määttä and Björkman 2024; Mitchell and Loughlin 2022). Studies investigating person‐centred care interventions on patient safety and clinical outcomes remain equivocal (Rossiter et al. 2020; Summer Meranius et al. 2020). Despite these drawbacks, educators and advocates of person‐centred care continue to rote champion its principles without fully acknowledging the considerable divide between the utopian rhetoric and day‐to‐day practice (McCrae 2013; Sobolewska et al. 2020). As a professional construct implemented by practitioners (Grover et al. 2022), person‐centred care paradoxically de‐centres the very patients it claims to prioritise. As we deconstruct the concept of person‐centred care, we argue that it is no longer a viable or coherent framework. We contend that person‐centred care is, in effect, ‘dead’; its demise orchestrated by those responsible for delivering it, or perhaps revealing that it never truly existed in the first place. In exposing the internal contradictions, we challenge the idea that it ever genuinely placed the patient at the centre of care. In the wake of our critique, we turn towards the possibilities that could emerge in a ‘post‐person‐centred care’ era. To this end, we begin our commentary by drawing on Friedrich Nietzsche's idea of the Übermensch (‘Over‐man’) as a philosophical departure point, returning to it at the end to reconceptualise healthcare practice through the figure of the ‘*Über‐professional’*. This individual acts from a position of *authenticity*, guided by their own values rather than values that are externally imposed by others. We propose that such a vision may circumvent many of the issues we have identified with person‐centred care.

## 2 Nietzsche's ‘Übermensch’

In his seminal work ‘*Thus Spoke Zarathustra: A Book for Everybody and Nobody*’ (Nietzsche 2003), Nietzsche narrates the wisdom of a fictional prophet‐like character ‘Zarathustra’, who through his adventures, serves as a vehicle for Nietzsche's philosophical ideas. In the story, Zarathustra descends from a mountain following a 10‐year self‐imposed seclusion, to share the wisdom he has learnt with humanity. His interactions however soon reveal a society whose human condition is mediocre, lacking in ambition, aspiration or creativity. Zarathustra realises most people are content with personal comfort and conformity resulting in an unchallenging existence. He concludes that herd mentality has ultimately stifled individual expression leading to a stagnant and unremarkable civilisation.

Nietzsche, confronting widespread cultural malaise, proposed a radical solution in the figure of the Übermensch (often translated as ‘Overman’ or ‘Superman’). This philosophical ‘remedy’ was the antithesis that aimed to transcend the condition of what he described as the ‘*last man*’, a group that Nietzsche saw as being representative of, and debilitated by, societal conformity, institutional thinking and the complacency of conventional wisdom. In contracts, the ‘Übermensch’ embodies a re‐evaluation of values, grounded in individual creativity, self‐mastery, and the pursuit of authentic living. Perceived as hierarchically superior to the last man, the Übermensch was in Nietzsche's words ‘*the lightning out of the dark cloud—man*’ (Nietzsche 2003, p. 31):

What is the ape to man? A laughing‐stock, a thing of shame. And just the same shall man be to the Superman: a laughing‐stock, a thing of shame.

(Nietzsche 2003, p.23)

To achieve the state of the Übermensch, Nietzsche narrates an allegorical tale of the journey of the ‘spirit’ through three transformative stages. The first stage, ‘the Camel’, symbolised the spirit's willingness to bear the burdens of life, embracing both its joys and hardships. From here, the spirit is transformed into ‘the Lion’, embodying the strength to challenge and defy external constraints and imposed values. And finally, the spirit evolves to become ‘the Child’, representing innocence, freedom, and the capacity for creative renewal, marking the beginning of a self‐driven cycle of growth and transformation.

Notably, Nietzsche's critique was primarily aimed towards dominant religious and moral systems. He argued these imposed restrictive values that stifled human creativity, individuality, and the fundamental internal driving force behind actions and behaviours, which he metaphorically termed ‘Will to Power’. For Nietzsche, a lack of ‘Will to Power’ signified weakness, diminished resolve and an aversion to risk‐taking. In contrast, the Übermensch embodied a robust ‘Will to Power’ which was realised through self‐actualisation, the pursuit of creative potential and the ability to transcend societal limitations to achieve a higher state of life‐affirming existence. A fulfilling and well‐lived life, according to Nietzsche, is one that is aligned with personal values and which was free from adverse societal influences and pressures. However, when in the story Zarathustra tries to convey his message of the Übermensch to the masses, he is met with mockery, realising most people are resistant to change and are ignorantly satisfied with the suppressing status quo.

For those who embrace the concept of the ‘Übermensch’ and a robust ‘Will to Power’, it offers a powerful mind‐set for personal growth and realising one's inner potential to overcome life's challenges. However, accepting Nietzsche's idea of the ‘Will to Power’ should not be misconstrued as a pursuit of personal power or domination at the expense of others; such a misinterpretation can lead to dangerous consequences. Nietzsche's philosophy and works were notoriously misappropriated and distorted by the Nazi regime to align with fascist ideology (Golomb and Wistrich 2009). Instead, the ‘Will to Power’ encourages individuals to take responsibility for their own lives, balancing personal empowerment with ethical responsibility to prevent exploitation or harm. Furthermore, this vision also invites a re‐evaluation of contemporary ideologies, such as person‐centred care, challenging not only the underlying purpose but also its practical application.

## 3 Illusions of Person‐Centred Care

In employing the Derridean strikethrough in the above immediate subheading, we signal through this gesture that the term ‘person‐centred care’ is simultaneously necessary yet inadequate. By placing ‘person‐centred care’ under erasure, we do not discard the term but rather bring attention to its fragility indicating that while it must be used, there are slippages in its meaning. Derrida's concept of *différance* further illustrates how the meaning of ‘person‐centred care’ is never fully present or conveyed, and is constantly deferred through differing interpretations and usages. This forms the basis of our argument that person‐centred care was never truly ‘alive’ to begin with; its existence is more illusory than substantive, marked by discrepancies in its conceptualisation, definition, interpretation, operationalisation, and application (Summer Meranius et al. 2020; Brione 2022). The literature acknowledges these deficiencies, most notably the numerous and ongoing attempts to define person‐centred care. Within these attempts, variability is evident in its proposed core elements, application in best practice guidelines, and criteria for evaluating its efficacy (Grover et al. 2022). From a literary perspective, the term is often employed interchangeably with related, yet non‐identical concepts to suit the rhetorical or ideological needs of the author. For example, terms such as *client‐centred care*, *individualised care*, *holistic care*, and *humanistic care* are all used as apparent synonyms; while the distinction between related notions like *shared decision‐making*, *concordance*, and the framing of the patient as consumer is not always clear (Håkansson Eklund et al. 2019; Latimer et al. 2017; Pieterse et al. 2023). It is this undecidability that the strikethrough attempts to reveal.

Complicating matters further, are its historical influences and traces. ‘Person‐centred care’ has its roots in psychology and humanist ‘client‐centred’ models that were developed in the 1950s (Edgar et al. 2020). Such traces may explain why ‘person‐centred care’ appears to have gained prominence in older adult care, shaped by contributions from nurses and psychotherapists, while competing ‘patient‐centred’ concepts have been more widely adopted by medical staff across all patient groups (Edgar et al. 2020). Furthermore, there are ongoing debates about what is actually meant by the word ‘centredness’ (Feldthusen et al. 2022) with considerable diversity in understanding leading to differences in its interpretation and application across healthcare settings and between different stakeholders (Määttä and Björkman 2024; Mitchell and Loughlin 2022). With such diversity of views, professional groups are reported to conveniently select or prioritise different aspects of ‘person‐centred care’ to promote their own interests, often to the detriment of the patient and particularly marginalised groups (Smith et al. 2022). As a result, determining which ‘person‐centred care’ approaches and strategies are effective within different health system contexts, pose significant challenges (Nolte et al. 2020). While some have embraced this degree of indeterminacy and vagueness arguing that what counts as being ‘person‐centred’ can vary across different care contexts (Mitchell et al. 2022), others have sought to reconceptualise ‘person‐centred care’ altogether (Tieu et al. 2022), or have proposed alternative approaches. One such model is ‘subjunctive medicine’ which involves co‐constructing temporary but purposeful clinician‐patient shared social worlds (Hardman and Ongaro 2020). Given this variability, the challenges of interpreting ‘person‐centred care’ become particularly evident when attempting to operationalise the term for teaching purposes. ‘Person‐centred’ educational interventions often focus on ideal behaviours, such as supportive, personalised, and coordinated care that promotes dignity, compassion, and respect. However, as others have argued, without a robust theoretical foundation or means to direct experiential knowledge, teaching can foster a reductionist understanding of ‘person‐centred care’ resulting in cognitive dissonance with the more dominant and privileged biomedical model (Bansal et al. 2022).

Returning to the illusionary nature of ‘person‐centred care’, we now question for what purpose this narrative was constructed and whose interests it serves. From a Foucauldian perspective, the discourse surrounding ‘person‐centred care’ could be viewed as functioning as a form of ‘pastoral power’, serving as a professional strategy to uphold patient discipline while appearing to promote patient agency. Under the guise of placing the ‘patient at the centre of care’, this narrative subtly reinforces professional authority, seeking to shape patient behaviour in ways that align with existing institutional norms and professional convenience. The power dynamics embedded in this discourse create an illusion of agency, while in reality, the structure and delivery of care remain controlled by health professions, institutions and policy‐makers (Brione 2022; Martin and Waring 2018; O'Dwyer 2013). More critically, the discourse surrounding ‘person‐centred care’ may perpetuate a cycle of governance, serving as a mechanism for regulating professional conduct and so legitimising the authority of stakeholders to continue to shape practices and policies according to vested interests (McGivern 2024). Much like the historical appropriation and distortion of Nietzsche's philosophy by the Nazi regime (Golomb and Wistrich 2009), the person‐centred discourse is similarly strategically mobilised as part of the broader apparatus of governance. Both cases underscore how language and philosophical ideas can be used by dominant systems, that on face value appear to empower and emancipate, but subtly regulate an individual's conduct and behaviour.

In short, we argue that the rhetoric of ‘person‐centred care’ largely operates as a mechanism of control, allowing professional bodies to present themselves as being politically correct while largely preserving the status quo. By adopting this language, professional bodies align themselves with the prevailing discourse of compassion and patient advocacy, which in turn helps to maintain their status and legitimacy. However, this veneer masks the deeper realities of entrenched power structures within healthcare systems. Thus, rather than genuinely shifting power to patients, the concept often functions as a tool for the professions to retain authority under the guise of progressive care. Similar critiques have also been made of related practices. For instance, the rhetoric around ‘client‐centeredness’ has become a central component of occupational therapy's professional self‐image and public discourse. However, when applied in practice, it has been criticised for its inconsistent adherence to these principles, but also for its use as a rhetorical device to promote self‐interest, enhance professional status and power (Whalley Hammell 2013). Others, also adopting a Foucauldian analysis, concluded that person‐centred care in pre‐pandemic contexts was a fallacy and that care delivery during COVID‐19 demonstrated it was at best fragile and at worst rendered invisible (Byrne et al. 2024). By recognising its illusionary nature and acknowledging its limitations, health professionals themselves can begin to contemplate the evident flaws within the ‘person‐centred care’ philosophy, especially when they personally observe and experience organisational work practices that contradict these principles.

## 4 The Fallacy of Person‐Centred Care

A statement attributed to Bertrand Russell captures the importance of questioning long‐held assumptions:

“In all affairs — love, religion, politics or business — it's a healthy idea, now and then, to hang a question mark on the things you have long taken for granted.”

(Bertrand Russell, quoted in Reader's Digest 1940)

We therefore take a closer examination of person‐centred care and suggest it is not merely illusory, but potentially fallacious, especially when viewed from the standpoint of the patient. While person‐centred care claims to place the patient at the ‘centre’ of care, the discourse and institutional structures that support it, including its language, frameworks, and methods of implementation, remain deeply professionalised. As such, we argue it is not a patient‐led model but rather a professional construct that dictates how interactions with patients ought to be conducted. From a Derridean perspective, this generates a sense of undecidability; a tension where both, and yet neither patient or professional appear to occupy the ‘centre’. This instability may explain in part the significant obstacles to the delivery of person‐centred care which persist across care settings and among different professional groups (Kitson et al. 2023; Murry and Desselle 2024; Kayes and Papadimitriou 2023). Person‐centred care remains more of a professional aspiration than a lived reality, as it is implemented within healthcare systems that were not originally intended to or designed in a way to support such a philosophy.

Further consequences of this undecidability can be seen through the frequently reported barriers that hinder person‐centred care practice. These include for instance, limited patient and professional capacity (e.g., knowledge and skills) to engage with person‐centred care (Liao et al. 2023; Da Costa et al. 2020; Byrne et al. 2024), organisational constraints (e.g., time limitations), as well as entrenched biomedical approaches (Phelan et al. 2020). It is evident these practices and systems, at all levels, have historically been structured around professional convenience, institutional norms, and bureaucratic processes that prioritise the efficiencies of healthcare providers over the individual needs of patients. Therefore, adopting a ‘person‐centred approach’ can become problematic at all levels of care. For example, practically at the patient level, rigid appointment scheduling and issues accessing services create barriers to care. Because these challenges disproportionately affect marginalised groups, they reveal a systemic failure to design care that is responsive to individual needs (Hui et al. 2020). At the provider level, existing reliance on technology, checklists and the dominance of evidence‐based medicine underscores a focus on efficiency and standardisation, often at the cost of meaningful, personalised patient engagement. Hospital policies for instance, with restricted visiting hours, further highlight the prioritisation of institutional convenience over the emotional and psychological well‐being of patients and their families. More broadly, such practices may reflect the Western professional individualistic conception of personhood, privileging self‐determination over non‐Western philosophical perspectives that emphasise familial involvement in care decisions.

Organisational targets, perverse incentives and transformation of healthcare into a commodity further hinder the prospects of promoting or achieving a person‐centred care practice (Mills et al. 2023; Khan et al. 2020). Specifically, neo‐liberal and corporate healthcare culture, with its strong emphasis on reimbursement‐driven models, has created both philosophical and ethical dissonance. This in turn, has led to the ‘illusion’ of delivering person or ‘client‐centred care’, where the ideals are overshadowed by financial and organisational priorities (Damarell et al. 2020). Others have commented on the ideological clash between person‐centred care and more privileged dominant paradigms, not more so than the scientifically‐led evidence‐based movement, which undermines experiential knowledge and the moral imperative of person‐centred care (Gupta and Taff 2015; Anjum 2016). Adopting a Hegelian perspective, it has been suggested that we have yet to even fully synthesise a person‐centred care approach as it remains embedded in the paradigm of the evidence‐based movement, which itself was a reaction to earlier less favourable practices based on professional judgements (Karunatilake 2021).

Broader contradictions also emerge in the context of healthcare labour relations. A particularly significant, yet often underexplored dilemma, concerns the viability of person‐centred care when healthcare professionals engage in industrial action or ‘job action’ in response to concerns over patient safety, poor working conditions or inadequate remuneration. While such action is legally and ethically justified in many cases, especially within failing healthcare systems (Essex and Weldon 2022; Essex et al. 2023), its potential disruption to patient care introduces a fundamental contradiction and cognitive dissonance. Healthcare professionals are expected to place the patient at the centre of care, yet industrial action, particularly strikes, are often designed to withhold services precisely to exert institutional pressure. Although evidence suggests that strike action rarely results in worsened patient outcomes or mortality (Essex et al. 2022), this data does not fully account for disruptions in care continuity, patient anxiety, or delayed access to treatment (Rickert 2023).

To further our point, when viewed from an ethical standpoint, person‐centred care offers no clear framework to navigate or legitimise such strike actions. Utilitarian reasoning might justify strikes if they lead to long‐term improvements for both staff and patients. In contrast, deontological ethics might reject any interruption in care, arguing that healthcare professionals have a non‐negotiable duty to serve their patients. It is this second ethical position that poses a conundrum and threat to person‐centred care introducing and exposing the fragility of the concept as a guiding principle in real‐world conflicts. The framework lacks the moral architecture to balance individual duties against collective resistance. It is at these junctures that person‐centred care risks becoming no more than a rhetorical device, conveniently invoked when seeking to maintain or elevate professional status, yet dismissed when its principles conflict with actions that undermine self‐interest. Under these circumstances, the fallacy of person‐centred care again becomes self‐evident suggesting that it can no longer be considered a viable guiding concept for healthcare professionals. In what we have termed a *post‐‘person‐centred care’ era*, a power vacuum will inevitably emerge allowing new and possibly more authentic models to gain traction, free from the veneer of professional or politically correct rhetoric.

## 5 The ‘Über‐Professional’: A Call to All and None

“You cannot enslave a mind that knows itself, that values itself, that understands itself.”

(Maathai 2008)

As prevailing conceptions of the ‘person’ in health sciences are being re‐evaluated (Holmes et al. 2024), we return to Nietzsche's ‘Übermensch’ to reimagine practice through the figure of the ‘Über‐professional’. This is an individual whose ‘*Will to Power’* enables them to transcend institutional constraints and conventional professional norms when these no longer align with the true essence of care. We envisage the Über‐professional could embody a more authentic mode of ‘health‐care’ practice, guided by self‐mastery and intellectual rigour, enabling them to champion patient advocacy in its fullest sense. Through this approach, the Über‐professional ensures that patient voices are not only heard but also genuinely respected, with their interests consistently represented in all care decisions. This more nuanced model thus marks a shift toward a higher standard of professional authenticity and ethical integrity, while reducing the risk of cognitive dissonance. For example, in returning to the issue of strike action, unlike the contradictions inherent in the ‘person‐centred care’ philosophy, the Über‐professional's decision to strike would be made with a greater sense of authenticity allowing their action to proceed free from emotional struggle. Although their decision may result in similar immediate consequences for patients, Über‐professionals are not constrained by values imposed by others. They may indeed choose to evaluate and mitigate any risks or encourage a collaborative approach with patients, but ultimately decide for themselves what actions, if any, are appropriate.

Unlike most frameworks typically taught in professional training, the journey toward Über‐professionalism would most likely begin with a deep critical reflection on how the discourse of ‘person‐centred’ care falls short of practitioners' own experiences and personal practice. In line with Nietzsche's step‐by‐step development framework, this introspection enables them to first embrace their present circumstances, initiating a process of resistance against ineffectual ideas, particularly the rhetoric and obligations surrounding supposed ‘person‐centricity’. By challenging herd mentality and conventional norms, they seek to uncover and address inconsistencies or biases within their own approach to care. Ultimately, they reach the ‘Child stage’, Nietzsche's metaphor for a state of renewal and freedom, where they cultivate a wiser and more independent mind‐set that transcends traditional role expectations and which embraces a more authentic individualised practice. More broadly, the Über‐professional's untethered curiosity, courage and honesty will offer new insights and liberate them from the constraints of wider inhibitory institutional guidelines, algorithms, and conventional work practices that fail to serve the patient. While we have attempted to remain faithful to Nietzsche's ideas, we do not strictly anticipate this to be such a linear process, but one that is more realistically iterative and perhaps even unpredictable.

Nevertheless, in this ongoing struggle, adaptability and self‐mastery become increasingly crucial as Über‐professionals pursue personal growth toward the ultimate goal of *authentic* practice in service of the patient. Crucially, this new approach neither signals a return to the paternalism of the past nor exacerbates the imbalance of ‘expert power’ between healthcare professionals and patients. The risk of such an imbalance is largely mitigated by the broader contemporary forces of consumerism, patient choice, and empowerment. Furthermore, we do not preclude the idea of *patients* as Über‐professionals, a natural evolution from patient, ‘expert patient’ to now Über‐professional. In the Nietzschean sense, the drive or ‘Will to Power’ refers to each parties internal striving for self‐overcoming, self‐actualisation, and the realisation of one's potential. We therefore envision patients and professionals as ‘Über‐professionals’, all resisting what they perceive as failures in healthcare as they forge authentic relationships and genuine connections with one another. This process has the potential to foster a more confident and empowered patient‐professional identity, grounded in their unique core values.

## 6 Concluding Remarks

Unlike Zarathustra's failed invitation to society to work towards the status of the Übermensch, our notion of the ‘Über‐professional’ may hold deeper significance in today's age of individualism and calls for a greater pragmatism in healthcare (Greenhalgh and Engebretsen 2022). For many, it may represent more than simply unlocking personal potential; it signifies emancipation. Just as Nietzsche's concept of the Übermensch was an antithesis to a stagnant and unremarkable human civilisation, the Über‐professional can similarly be viewed as a dialectical response to the flawed notion of ‘person‐centred care’. The departure from familiar modes of operation will inevitably face peer‐to‐peer scepticism, organisational resistance, and institutional opposition. Yet, it is precisely this willingness to confront healthcare conditioning, propaganda and even hypocrisy that distinguishes Über‐professionals from the herd and underscores their commitment to authenticity. Their struggle may persist until the growing contradictions within ‘person‐centred care’ and similar frameworks become more evident and undeniable, ultimately leading to a paradigmatic shift.

Without wishing to fall prey to our own analysis, this paper should caution against the assumed fixedness or absoluteness of any such concepts in the Derridean sense. We humbly acknowledge that any ‘Über‐professional’ model may require testing, further refinement and synthesis over time, and yet also could ultimately be seen just as fallible as the concept it seeks to challenge. It is a reminder of the inevitable slippages that occur in meanings, their evolving and deferred nature. However, in the present context, it is our opinion that the acute ‘treatment’ for the widespread uncritical acceptance of ‘person‐centred care’ is for patients and health professionals to cultivate a stronger ‘Will to Power’. It is a path reserved for those prepared to challenge convention and embrace change, *not* for personal gain, but to actualise this power in service of attaining greater well‐being. The initial step toward this enlightened future requires an honest and self‐reflective discussion on the teaching and actual practice of ‘person‐centred care’, alongside broader research to explore which patients or healthcare professionals, if any, are inclined to pursue this more authentic approach. This reflection could also extend to a broader critique of how social institutions manipulate selective narratives to construct and circulate ‘truth’. The benefits of an Über‐professional mentality, as we conceptualise it may help us all to more effectively challenge dominant post‐truth political and media narratives, expose and fight oppression, and advance a commitment to social justice within and beyond healthcare.

## Conflicts of Interest

The authors declare no conflicts of interest.

## Data Availability

Data sharing not applicable to this article as no datasets were generated or analysed during the current study.
